# 773 Insurance Coverage Does Not Increase Risk of Depression and Anxiety in Burn Patients

**DOI:** 10.1093/jbcr/irac012.326

**Published:** 2022-03-23

**Authors:** Laura Pezzopane, Anthea Gray, Alfredo C Cordova, Katherine Bergus, Nicole O Bernal

**Affiliations:** The Ohio State University, Columbus, Ohio; The Ohio State Medical Center, Columbus, Ohio; The Ohio State University Wexner Medical Center, Dublin, Ohio; The Ohio State University, Columbus, Ohio; The Ohio State University Wexner Medical Center, Columbus, Ohio

## Abstract

**Introduction:**

A burn injury can have long-term mental and physical effects on individual patients. When burn injuries occur at work, there is an additional unfamiliar stress of income loss and dependence on a third-party payer. Patients with claims through the Bureau of Worker’s Compensation (BWC) report frustration and overall dissatisfaction in working with the BWC to achieve claim coverage. Of workers who are off of work more than 5 days due to a work-related injury, 10% are diagnosed with depression in the 12 months following the injury (Carnide, 2016).

In our clinic, screening for depression and anxiety is done through the Patient Health Questionnaire (PHQ-4), a valid four-item assessment tool that utilizes a Likert style measurement to assess symptoms of depression and anxiety (Kroenke et al., 2009). This is a health questionnaire that determines a patient’s risk for depression and anxiety as mild, moderate, or severe.

**Objective:**

To determine if there is a correlation between insurance type and PHQ-4 scores in burn patients. We hypothesized that risk of depression and anxiety could differ based on payer, which can affect a patient’s access to care, referral approval, and financial burden of treatment. Additional focus was placed on BWC patients due to their reported frustrations and dissatisfaction in working with BWC and the established correlation between depression and missed work.

**Methods:**

A quality improvement project was initiated based on increased rates of referrals for psychological evaluation and treatment in BWC patients. A retrospective review was conducted of outpatient burn clinic visits where a PHQ-4 questionnaire was completed in the past 3 fiscal years: 7/1/2019-6/30/2021.

**Results:**

Total of 1932 visits with PHQ-4 collection were reviewed within the above specified time frame. The rates of moderate and high-risk scores for anxiety and depression were highest with BWC and Medicaid patients. Patients with private insurance showed a lower risk of moderate and severe depression. However, there was no significant difference when comparing BWC verses all other insurance. Table 1

**Conclusions:**

Overall there was no significant difference in risk of anxiety and depression with BWC versus other insurance coverage based on PHQ-4 scores. Limitations of the study include no distinction of extent of burn injury/burn depth, burn care/treatments, length of hospital stay if any; no distinction was made amongst BWC patients and their length of time off of work. A portion of the timeframe reviewed was during the COVID-19 pandemic.

